# Insights into the structure and function of the histidine kinase ComP from *Bacillus amyloliquefaciens* based on molecular modeling

**DOI:** 10.1042/BSR20220352

**Published:** 2022-10-28

**Authors:** Lulu Wang, Ruochen Fan, Zhuting Li, Lina Wang, Xue Bai, Tingting Bu, Yuesheng Dong, Yongbin Xu, Chunshan Quan

**Affiliations:** 1 School of Life Science and Biotechnology, Dalian University of Technology, No. 2 Linggong Road, Dalian 116024, Liaoning, China; 2 Key Laboratory of Biotechnology and Bioresources Utilization of Ministry of Education, College of Life Science, Dalian Minzu University, China; 3 Department of Bioengineering, College of Life Science, Dalian Minzu University, Dalian 116600, Liaoning, China; 4 Institute of Cancer Stem Cell, Dalian Medical University, 9 Western Lvshun Road, Dalian 116044, Liaoning, China

**Keywords:** Bacillus amyloliquefaciens Q-426, histidine kinase ComP, kinase activity, phosphotransferase activity, structure prediction

## Abstract

The ComPA two-component signal transduction system (TCS) is essential in *Bacillus* spp. However, the molecular mechanism of the histidine kinase ComP remains unclear. Here, we predicted the structure of ComP from *Bacillus amyloliquefaciens* Q-426 (BaComP) using an artificial intelligence approach, analyzed the structural characteristics based on the molecular docking results and compared homologous proteins, and then investigated the biochemical properties of BaComP. We obtained a truncated ComP_S_ protein with high purity and correct folding in solution based on the predicted structures. The expression and purification of BaComP proteins suggested that the subdomains in the cytoplasmic region influenced the expression and stability of the recombinant proteins. ComP_S_ is a bifunctional enzyme that exhibits the activity of both histidine kinase and phosphotransferase. We found that His571 played an obligatory role in the autophosphorylation of BaComP based on the analysis of the structures and mutagenesis studies. The molecular docking results suggested that the HATPase_c domain contained an ATP-binding pocket, and the ATP molecule was coordinated by eight conserved residues from the N, G1, and G2 boxes. Our study provides novel insight into the histidine kinase BaComP and its homologous proteins.

## Introduction

The proteins comprising the two-component signal transduction systems (TCSs) are ubiquitous phosphorelay signal transduction proteins that play a crucial role in sensing and responding to diverse extracellular stimuli [1–3]. TCSs have been recorded in almost all domains of life, including prokaryotes, fungi, and plants [4]. However, TCSs are conspicuously absent in animals, making them promising novel drug targets [5]. The TCSs are generally composed of histidine kinase (HK) and the response regulator (RR) and accomplish signal transduction through the phosphorylation of the RR by HK [3,6,7]. The HK undergoes a conformational change after sensing an extracellular stimulus, then it autophosphorylates at a conserved histidine residue using ATP and generates the phosphate group [6,8,9]. The phosphate group can then be transferred to the aspartate residue in RR; after that, the HK initiates a response by regulating the expression of the target genes [6,8,9].

ComQXPA is a vital quorum sensing (QS) system in *Bacillus amyloliquefaciens* that controls the synthesis of lipopeptides, such as bacilomycin D, fengycin A, fengycin B, and surfactin [10–12]. The ComQXPA system consists of the isoprenyl transferase ComQ, the signal peptide ComX, and the HK ComP, the RR ComA [13,14]. In this system, the ComX prepeptide signal is cleaved and prenylated by ComQ to generate the pheromone ComX [15,16]. Once modified, this pheromone binds to and activates ComP, which subsequently phosphorylates the response regulator ComA [17]. The activated form of ComA is necessary for the transcription of the *srf* operon, which is essential for the production of surfactin [18,19]. ComP is an essential member and plays a vital role in signal transduction across the cellular membrane in the ComPA TCS [13,20]. It is known that membrane proteins are notoriously difficult to study due to their partially hydrophobic surfaces, flexibility, lack of stability, and low expression [21]. The expression and purification of ComP are challenging because it is a multitransmembrane protein with a high molecular weight and structural information on ComP is lacking [22]. Therefore, the structural characteristics and molecular mechanism of ComP remain unknown.

Fortunately, artificial intelligence (AI) has proven to have tremendous potential in several areas of health care, including the prediction of the 3D structures of proteins [23]. The AI approach can predict the functional, folded structure of a protein molecule based on its amino acid sequence, right to the position of each atom in 3D space [24]. Three methods are mainly used in the software to predict the 3D structure of a protein, including homology modeling, protein threading, and *de novo* methods [25]. Homology modeling is a method that constructs a model of the ‘target’ protein from its amino acid sequence and an experimental 3D structure of a related homologous protein; some examples include the SWISS-MODEL [26], Phyre^2^ [27], FoldX [28], and ESyPred3D [29]. Protein threading is a protein modeling method and is used to model proteins that, despite containing the same folds as proteins with known structures, do not exhibit the homologous protein structures; some examples include HHpred [30], RaptorX [31], and IntFOLD [32]. However, it was challenging to predict the tertiary structure of full-length ComP through homologous modeling or protein threading, as few known structures of full-length homologous proteins are available in the Protein Data Bank (PDB). The *de novo* method is an algorithmic process by which the protein 3D structure is predicted based on its amino acid sequence; some examples include ROBETTA [33], trROSETTA [34], I-TASSER [35], tFold [36], and AlphaFold2 [37]. AlphaFold2 is a computational approach that is capable of predicting 3D structures of proteins to nearly experimental accuracy in a majority of cases [37]. An AI approach such as AlphaFold will accelerate the advancement of structural bioinformatics and may become an essential tool in modern biology [37].

In the present study, we predicted the tertiary structures of ComP from *B. amyloliquefaciens* Q-426 (BaComP) using AI through AlphaFold2. Based on the predictions, we constructed several recombinant plasmids containing the *comP* gene of the cytoplasmic region, ComP_S_ (residues 563–763), which were successfully expressed and highly purified. We observed that the recombinant ComP_S_ exhibited both histidine kinase and phosphotransferase activity. The mutagenesis study revealed that His571 played a crucial role in the kinase activity of ComP_S_. Our study provides novel insights into the current understanding of BaComP, and the detection methods described in our study may provide novel methods for future experiments to detect activities of other HKs.

## Materials and methods

### Prediction of the secondary and tertiary structures of BaComP

The secondary structure of BaComP was analyzed by nine online software programs, including CCTOP (https://octopus.cbr.su.se/), HMMTOP (http://www.enzim.hu/hmmtop/), Octopus (https://octopus.cbr.su.se/) [38], Philius (http://www.yeastrc.org/philius/pages/philius/runPhilius.jsp) [39], Phobius (https://phobius.sbc.su.se/) [40], Pro [41], Prodiv [41], Scampi [42], and ScampiMsa (https://scampi.bioinfo.se/) [43]. The topological structure of BaComP was predicted by Protter (http://wlab.ethz.ch/protter/#) [44], an open-source tool for the interactive integration and visualization of annotated and predicted protein sequence features, together with experimental proteomic evidence. The tertiary structure of BaComP was predicted by AlphaFold2, an AI method to predict protein structures that were highly successful in recent tests [37]. The molecular docking studies were carried out using HADDOCK 2.4 [45,46]. All figures representing structures were prepared using PyMOL [47].

### Construction of the expression plasmids

The strain *B. amyloliquefaciens* Q-426 was isolated and purified from compost samples at our laboratory in Dalian, China. The primers were designed based on the sequence of BaComP (NCBI: AWV55524.1) and are listed in Table 1. The gene encoding BaComP was amplified from the genomic DNA of *B. amyloliquefaciens* Q-426 by polymerase chain reaction (PCR). Then, the amplified fragments were inserted into the pPROEXHTa vector (Invitrogen, U.S.A.), and pPROEXHTa-ComP was transformed into *Escherichia coli* BL21 (RIL) cells, which were then cultured in Luria-Bertani (LB) containing 100 µg ml^–1^ ampicillin at 37°C overnight.

**Table 1:** The primers of ComP that were used in PCR reactions

Primers	Sequences (5′ to 3′)
F-BaComP_L_	GGGCCATGGATAAAGAAATACTCGATTTCCGG
F-BaComP_508–763_	GGGCCATGGATCGTGAAGAAATTTCTTGG
F-BaComP_513–763_	GGGCCATGGATTGGTTAAAAACATTATCT
F-BaComP_518–763_	GGGCCATGGATTCTTTTTACGTTAATGTT
F-BaComP_548–763_	GGGCCATGGATCCGGTCTGGCTCAAAAAG
F-BaComP_S_	GGGCCATGGATCGTTCGGATTTGGCCCGT
R-BaComP	GGGCTCGAGTTACAATTCCATTTCAATATCCGC

### Expression and purification of the recombinant proteins

The cells were further cultured in 1.0 L of LB medium containing 100 µg ml^–1^ ampicillin at 37°C until the OD_600_ reached 0.6–0.8. Then, 0.5 mM isopropyl-β-D-thiogalactoside (IPTG) was added, and the cells were incubated for 8 h at 30°C. The cells were then harvested by centrifugation and resuspended in lysis buffer containing 20 mM Tris (pH 8.0), 150 mM NaCl, 2 mM β-mercaptoethanol (β-ME), and 10% (v/v) glycerol. Next, the cells were disrupted by sonication for 20 min on ice and centrifuged at 13000 rpm at 4°C for 30 min. The supernatant was then mixed with Ni-NTA affinity resin (GE Healthcare, U.S.A.) and incubated for approximately 30 min on ice. Subsequently, the resulting slurry was loaded onto a column and washed using 200 ml of washing buffer containing 20 mM Tris (pH 8.0), 150 mM NaCl, 2 mM β-ME, 10% (v/v) glycerol, and 30 mM imidazole. The recombinant ComP with the 6 × His tag was eluted with 30 ml washing buffer supplemented with 250 mM imidazole. Then, the eluted fractions were analyzed by sodium dodecyl sulfate-polyacrylamide gel electrophoresis (SDS-PAGE) on a 15% gel and stained with Coomassie blue. The target protein was concentrated using Centriprep columns (Millipore, U.S.A.) with a buffer containing 20 mM Tris-HCl (pH 8.0), 150 mM NaCl, 2 mM β-ME, and 10% (v/v) glycerol.

### Circular dichroism (CD) spectroscopy

CD spectroscopy was performed to detect the structural integrity of ComP using a chirascan II spectropolarimeter (AppliedPhotophysics, U.K.). The proteins were centrifuged at 13000 rpm for 10 min at 4°C, and the supernatant was diluted to 0.1 mg ml^–1^ in 20 mM HEPES (pH 7.5) buffer containing 150 mM NaCl. The protein concentration was measured based on the ultraviolet (UV) absorption of the Tyr and Trp residues of the protein at 280 nm. The absorption spectra were recorded on a chirascan II spectropolarimeter at 4°C. The wavelength ranged from 190 to 260 nm with a step size of 1 nm and a bandwidth of 1 nm. Three consecutive scans were performed, and the mean was calculated, and the solvent signal was subtracted from all the spectra. The scans were averaged for percentage composition of helix, antiparallel, parallel, β-turn, and unordered structures with the software package CDPro. The *T*_m_ values of ComP_S_ in the absence and presence of ATP were detected using the chirascan II spectropolarimeter. The wavelength ranged from 200 to 260 nm with a step size of 1 nm, a bandwidth of 1 nm, a pathlength of 10 mm, time per point of 0.15 s, the degree ranged from 20 to 96°C, and the temperature of the samples was increased by 1°C per minute.

### Western blot analysis of the ComP_S_

In this paper, we used Ab-1 and Ab-3 antibodies to detect phosphorylated ComP_S_. These two antibodies, which could specifically recognize phosphorylated histidine kinases, were synthesized by our group [48]. The reaction system comprised 30 µg of protein, which was incubated with 5 mM ATP, 5 mM MgCl_2_, 10 mM β-Me, 20 mM HEPES, and 150 mM NaCl at 37°C for 30 min. The negative controls were BSA and ComP_S_ protein without ATP. The samples were boiled, resolved by 15% SDS-PAGE, and then blotted onto a polyvinylidene difluoride (PVDF) membrane at 80 mA for 45 min. Then, the membranes were blocked using 1× TBS buffer (20 mM Tris HCl, pH 8.0, 150 mM NaCl), 0.05% (v/v) Tween 20, and 1% bovine serum albumin (BSA) for 2 h at 22°C. The two membranes were incubated with antibodies Ab-1 (1:3000) and Ab-3 (1:3000) in blocking buffer at 20°C for 2 h. The membranes were then incubated with HRP-conjugated goat anti-rabbit IgG antibody (Sangon Biotech) (1:10000) for 2 h at 22°C. In the present study, we used the Tanon™ High-sig ECL western blotting Substrate kit (Tanon) to detect the samples; the membranes were incubated in the ECL substrate for 3 min and then captured using a Tanon 4600 chemiluminescent imaging system (Tanon, Shanghai, China).

### *In vitro* phosphotransferase assays of the ComP_S_

The phosphotransferase assay of the ComP_S_ was measured using the Kinase-Glo® Luminescent Kinase Assay Kit [49,50] and Western blotting [51]. The assay was performed in white-colored 96-well plates containing 0.125 µM ComP in the kinase reaction mixture, and the mixture was incubated at 37°C for 30 min. Then, a concentration gradient of ComA (4, 8, 16, 32 µM) was added (the negative controls did not contain ComA), and the mixture was incubated at 37°C for 10 min. Then, 50 µl of ATP detection reagent was added to the assay plates and incubated at 37°C for 10 min. The RLU signal was measured using the Synergy2 Multi-Mode Microplate Reader. All experiments were performed in triplicate. Phosphotransferase activity was measured by the reduction of thiophosphorylated ComP_S_ [52]. The phosphorylation state of ComP_S_ was detected using ATP-γ-*S* and a primary antibody specific for the alkylated thiophosphate ester, which was alkylated by alkylation reagent PNBM [51]. The ComP_S_ at 40 µM was autophosphorylated with 400 µM ATP-γ-*S* in the kinase reaction buffer for 30 min. A fraction of the phosphorylated ComP_S_ sample was used as a control before incubation with the receive domain of response regulator ComA (ComA_RD_). Then, the cells were incubated with 120 µM ComA_RD_ at RT for the designated time points. The reactions were stopped with 20 mM EDTA and then alkylated by 2.5 mM PNBM for 1 h at RT. The samples were separated by 15% SDS-PAGE and then transferred to PVDF membranes. Western blotting was performed as described above. The PVDF membrane was examined using an anti-thiophosphate ester antibody at 1:5000, which was detected with an HRP-conjugated goat anti-rabbit IgG antibody at 1:10,000. Band intensities for the phosphorylated ComP_S_ were quantified using ImageJ software and were normalized to that at control.

### Mutagenesis studies of ComP

The plasmids for the expression of the ComP_S_ mutants (ComP_H571A_, ComP_H625A_, and ComP_H571A/H625A_), which were used in the analysis of kinase activity, were constructed using two subsequent rounds of PCR. The primers were designed based on the sequence of BaComP (NCBI: AWV55524.1) and are listed in Table 2. The first round of PCR was used to amplify the upstream-mutated segment using the forward primer R-BaComP_H571A_ and the reverse primer F-BaComP_S_. The first round of PCR was also used to amplify the downstream-mutated segment using the forward primer F-BaComP_H571A_ and the reverse primer R-BaComP. The second round of PCR introduced an overhang using the DNA fragments generated in the first round of PCR as templates and primers F-BaComP_S_ and R-BaComP, which were cloned into the expression vector pPROEX-HTa (Invitrogen, U.S.A.) at the NcoI and XhoI restriction sites. Furthermore, the plasmids were transformed into *E. coli* BL21 (DE3) cells, which were cultured in LB medium. The plasmids pPROEXHTa-ComP_H625A_ and pPROEXHTa-ComP_H571A/H625A_ were constructed using the same method as the pPROEXHTa-ComP_H571A_. The ComP_S_ mutants were purified under the same conditions as the wild-type ComP_S_ protein.

**Table 2:** The primers of ComP_S_ mutants that were used in the PCR reactions

Primers	Sequences (5′ to 3′)
F-BaComP_H571A_	GCCCGTGATCTGGCAGATTCAGTGCTG
R-BaComP_H571A_	CAGCACTGAATCTGCCAGATCACGGGC
F-BaComP_H625A_	CGGGAGACGTGCGCAGAGCTTCGTCCT
R-BaComP_H625A_	AGGACGAAGCTCTGCGCACGTCTCCCG

## Results

### Secondary and tertiary structure prediction of BaComP

**Figure 1:**
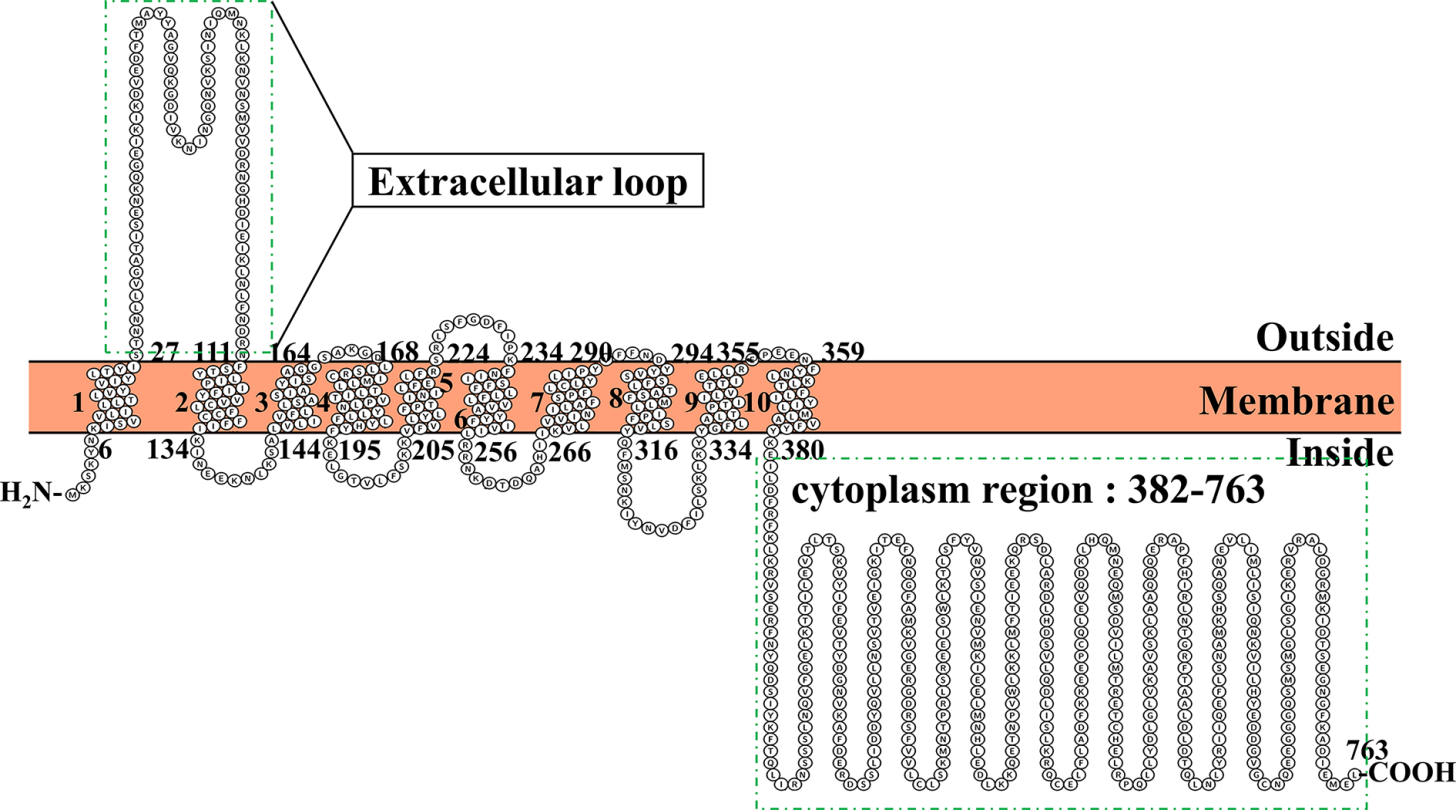
The schematic structure of BaComP topology The membrane protein topology of BaComP has ten transmembrane spanning helices and internally oriented N- and C-termini. The extracellular and cytoplasmic sides of the membrane are labeled at the beginning and end of each membrane helix, illustrated with a number indicating the residues of the sequence. The extracellular loop and cytoplasm region are marked by green boxes.

In the absence of a 3D structure for BaComP, we analyzed the transmembrane helices and the topological structure of BaComP using various programs available on the internet, including CCTOP [38], HMMTOP, Octopus, Philius [39], Phobius [40], Pro [41], Prodiv [41], Scampi [42], ScampiMsa [43], and Protter [44]. Based on the analysis of the prediction results, both the N-terminus and C-terminus of BaComP were located in the cytoplasm. A transmembrane domain was present at the N-terminal region that consisted of ten transmembrane helices, forming five extracellular loops and four intracellular loops. The first extracellular loop was sizeable, consisting of 85 amino acids (residues 27-111) that were located between the first and second helices (Figure 1 and Supplementary Figure S1). Moreover, we observed a large cytoplasmic region (residues 382-763) at the C-terminal (Figure 1). To verify the detailed information of these two domains, the 3D structure of BaComP needs to be analyzed.

**Figure 2:**
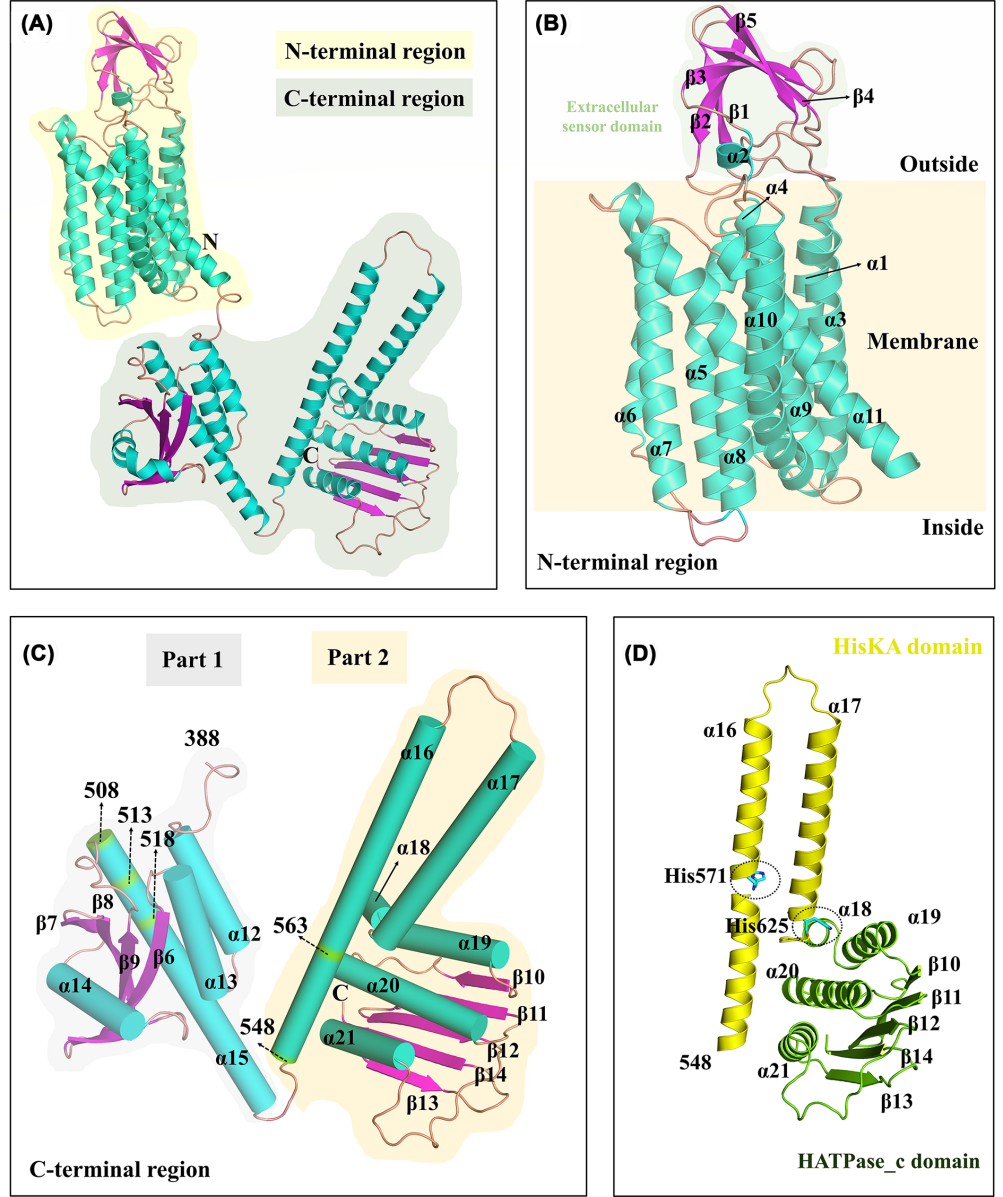
Folding patterns of the BaComP molecule as shown in the cartoon (**A**) The overall folding of the BaComP molecule is shown in the cartoon. The tertiary structure of BaComP was predicted by AlphaFold2, the α-helices and the β-strands were numbered, and the terminals were labeled. (**B**) The overall folding of the N-terminal region in BaComP. The extracellular sensor domain is marked in a black-dotted box. (**C**) The overall folding of the C-terminal region in BaComP. (**D**) The overall folding of Part 2 from the C-terminal region in BaComP. The His residues that can be potentially phosphorylated are shown as sticks.

To better understand the functions and mechanisms of BaComP, we predicted the overall structure of BaComP using AlphaFold2 [37]. The result is presented in Figure 2A, and BaComP consisted of an N-terminal region (residues 1-387) and a C-terminal region (residues 388-763). The N-terminal region comprised ten transmembrane segments (TMSs), which was consistent with the predictions of the secondary structure (Figure 2B). In previous studies, the number of TMSs in ComP was conflicting. Piazza first reported that BaComP had eight TMSs [22], while Mascher summarized that ComP-like proteins had eight to ten TMSs based on sequence analysis [53]. Determining the number of TMSs is essential to understand the structural and functional relationships of BaComP. In the present study, the prediction results of BaComP indicated that it contained ten TMSs, but further studies are still needed to determine the accurate structure of BaComP.

Our prediction study indicated that BaComP has a large extracellular region (residues 30-108) between the first and second transmembrane helices. The large extracellular region contains one α-helix (α2) and five β-sheets (β1-β5). To compare the 3D structure of the large extracellular region against those in the PDB and better understand the function, we performed a structural homolog search using the DALI server [54]. The results indicated that the large extracellular region of BaComP had structural similarity with the PDZ domain of several proteins, including the protease CtpB from *Bacillus subtilis* (BsCtpB, PDB code: 4C2E, *Z* score: 9.9, RMSD of PDZ domain: 1.011 Å) [55], the aminopeptidase N-family protein Q5QTY1 from *Idiomarina loihiensis* (IlQ5QTY1, 4FGM, 9.4, 0,786 Å), the serine protease HtrA3 from *Homo sapiens* (HsHtrA3, 4RI0, 9.3, 2.051 Å) [56], the DegS from *E. coli* (EcDegS, 1TE0, 9.1, 1.102 Å) [57], and the AlgW protein from *Pseudomonas aeruginosa* PAO1 (PaAlgW, 7CO7, 9.1, 1.130 Å) [58]. The superimposition results of these structures suggested that the large extracellular region of BaComP was consistent with the overall folding of the PDZ domain (Supplementary Figure S2A). The sequence alignment of BaComP, BsCtpB, IlQ5QTY1, HsHtrA3, EcDegS, and PaAlgW showed that the PDZ domains of these proteins shared low sequence identities at 21.95%, 18.92%, 24.24%, 10.96%, and 16.83%, respectively (Supplementary Figure S2B). Previous studies have reported that the PDZ domain of HKs is related to signal recognition and binding [53,59]. The low sequence identity of the PDZ domains might be due to the abundance and diversity of substrates [60]. These results suggested that the PDZ domain of BaComP may be bound to the signal peptide ComX, but the detailed mechanism remains unclear. Therefore, a complex structure is needed to understand the structural basis of ComX recognition by the BaComP PDZ domain.

In the predicted 3D structure of BaComP, the C-terminal region (residues 388-763) consisted of Part 1 (residues 388-543) and Part 2 (residues 549-763), and the two parts were connected by a short linker (residues 544-548) (Figure 2C). In Part 1, we observed one short α-helix (α12) and one long α-helix (α15) connected by a subdomain that contained a central four-stranded β-sheet (β6-β9) surrounded by loops and two α-helices (α13 and α14) (Figure 2C). In Part 2, we observed two long α-helices (α16 and α17), which were followed by a subdomain consisting of five β-sheets (β10-β14) and four α-helices (α18-α21) (Figure 2C). The sequence and structural analysis of the homologous proteins suggested that α16 and α17 of BaComP formed the HisKA (histidine kinase A) domain, followed by the HATPase_c (histidine kinase-like ATPase catalytic) domain located at the C-terminal (Figure 2D). These structural characteristics in BaComP were consistent with other typical HKs and might have similar functions, although no related studies on BaComP have been reported. Therefore, to analyze the structural–functional relationship of the HisKA domain and HATPase_c domain of BaComP, we dissected these two domains in this study.

### Dissection of the functional domains of BaComP

**Figure 3:**
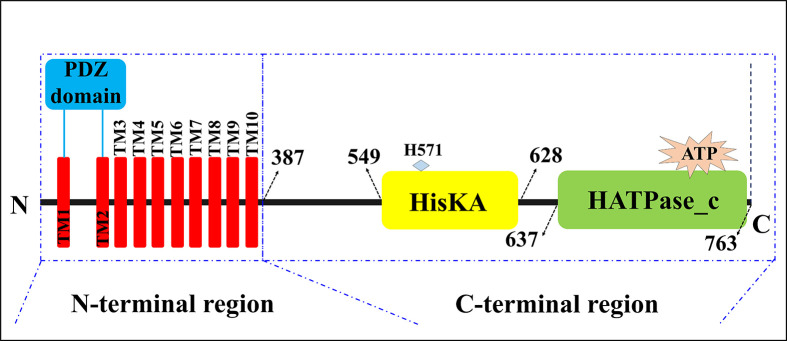
The schematic representation of the secondary structure prediction of BaComP The colored boxes represent the following characteristic structures: the transmembrane helix (red), the PDZ domain (blue), the HisKA domain (yellow), the HATPase_c domain (green), and the histidine site His571 (light blue).

In the present study, we analyzed two functional domains of BaComP, including the HisKA domain and the HATPase_c domain, and proposed a schematic diagram of BaComP (Figure 3). The HisKA domain is also named the DHp (dimerization and histidine phosphorylation) domain, while the HATPase_c domain is also named the CA (catalytic and ATP-binding) domain [6,61]. We analyzed the structural characteristics of BaComP with its homologous proteins, including HK0853 from *Thermotoga maritime* (TM0853, PDB code: 2C2A) [8], the osmolarity sensor protein EnvZ from *Thermotoga maritima* MSB8 (TmEnvZ, 4KP4) [7], the sensor HK CpxA from *Escherichia coli* (EcCpxA, 5LFK) [62], the sensor protein WalK from *Lactobacillus plantarum* JDM1 (LpWalK, 4U7N) [63], the putative HK CovS from *Streptococcus mutans* UA159 (SmCovS, 4I5S) [64], HK CckA from *Caulobacter vibrioides* (CvCckA, 5IDJ) [65], and the sensor protein SrrB from *Staphylococcus aureus* (SaSrrB, 6PAJ) [66]. The results of the structural analysis indicated that all the abovementioned HKs contained the HisKA and HATPase_c domains at the C-terminus (Supplementary Figure S3). The results of superimposition of the HisKA domain suggested that BaComP had high RMSD values with TM0853 (10.351 Å), TmEnvZ (17.633 Å), EcCpxA (8.142 Å), LpWalK (4.744 Å), SmCovS (5.716 Å), CvCckA (3.818 Å), and SaSrrB (6.079 Å). Nevertheless, the overall structure of the HisKA domain of BaComP was similar to those of canonical HKs, as both are composed of two long α-helices (Supplementary Figure S3). The high RMSD values might be caused by the different lengths and angles of the two long α-helices. Based on the superimposition of the HATPase_c domain, BaComP exhibited high structural similarity with TM0853 (RMSD: 1.468 Å), TmEnvZ (3.585 Å), EcCpxA (1.634 Å), LpWalK (1.821 Å), SmCovS (1.896 Å), CvCckA (1.636 Å), and SaSrrB (2.007 Å). These results demonstrated that the structure of the HATPase_c domain in BaComP was highly conserved with homologous proteins that were composed of mixed α/β sandwich folds.

The results of sequence alignment indicated that BaComP (HisKA domain and HATPase_c domain) shared sequence identity with TM0853, TmEnvZ, EcCpxA, SaSrrB, LpWalK, SmCovS, and CvCckA, with values of 31.96%, 29.68%, 33.33%, 32.42%, 35.62%, 28.31%, and 29.68%, respectively (Supplementary Figure S4). Normally, the HisKA domain of typical HKs has a conserved H box (HELRTP) located at the first long α-helix, wherein the HKs can autophosphorylate on a histidine residue [61,67,68]. Interestingly, we observed two putative H-boxes (H-Box 1 and H-Box 2) in the sequence of BaComP (Supplementary Figure S4). The two H-boxes included two histidine residues, His571 and His625, which were located at H-Box 1 (HDSVLQ) and H-Box 2 (HELRPQ), respectively (Supplementary Figure S4). In the predicted structure of BaComP, His571 and His625 were located at α16 and α17 of the HisKA domain, respectively (Figure 2D). To confirm phosphorylation at histidine residues, we mutated His571 and His625 to alanine and detected histidine kinase activity (data see below).

The HATPase_c domain of typical HKs could bind to the ATP molecule, after which a phosphate group was transferred to the exposed histidine residue in the HisKA domain [69]. Previous studies have suggested that the HATPase_c domain contains conserved motifs, such as the N-box (ExxxNxxDA, wherein x is any amino acid), G1-box (DNGxGx), G2-box (GxxGxxxxS), and G3-box (Tx_n_GT) [70–72]. Interestingly, our sequence alignment analyses suggested that BaComP had three conserved boxes, including a putative N-box (681EFLSNAMKHS690), a putative G1-box (712DDGVGC717), and a putative G2-box (726SMSMGLSG733) (Supplementary Figure S4). To verify the binding mechanism of the HATPase_c domain of BaComP and ATP, we performed molecular docking using HADDOCK 2.4 [45,46]. We observed an ATP-binding pocket in the HATPase_c domain of BaComP, which consisted of two loops (loop 1-2) and the upper part of two α-helices (α20 and α21) (Supplementary Figure S5A). Interestingly, the putative N-box of BaComP formed loop-1 (691QAN693, between β11 and α20) and the upper part of α20, while the putative G1-box and G2-box of BaComP formed loop-2 (713DGVGCNQEEGGGQSMS728, between β12 and α21) and the upper part of α21 (Supplementary Figure S5A). These analyses demonstrated that the ATP-binding pocket contained the three highly conserved boxes in the HATPase_c domain of BaComP. In addition, we found an ATP molecule at the ATP-binding pocket, which exhibited a bent conformation and was stabilized by eight residues: Asn685, Lys688, His689, Gly716, Cys717, Ser728, Met729, and Gly730 (Supplementary Figure S5A). Furthermore, we also observed that three highly conserved residues, Ans685, Gly716, and Gly730, were involved in ATP binding. To better understand the ATP-binding mechanism, we compared the structural insights of BaComP with its homolog protein TM0853, which was bound to an ADP molecule (PDB code: 2C2A) (Supplementary Figure S5B). In the crystal structure of TM0853, the ADP molecule was located in the ATP-binding pocket similar to that in BaComP and was coordinated by eight residues (Asn376, Asn380, Lys383, Tyr384, Asp411, Arg430, Val431, and Leu446) from the N-box, G1-box, and G2-box of TM0853 [73]. These analyses suggested that the binding mechanism of ATP in BaComP was consistent with that of TM0853, and the putative N-box, putative G1-box, and putative G2-box may play crucial roles in ATP binding.

### Purification and biochemical characterization of the recombinant ComP proteins

**Figure 4:**
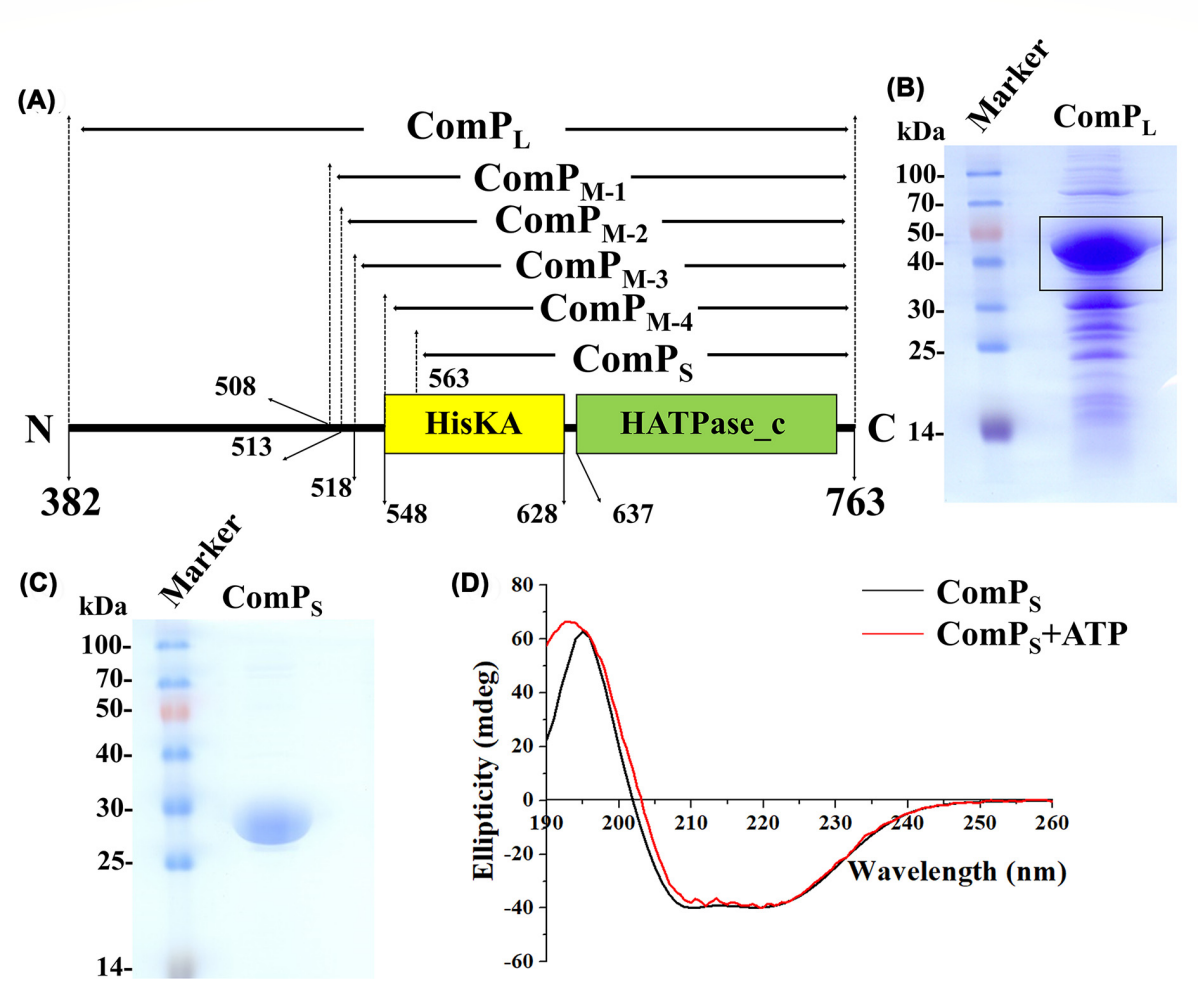
The purification results of the recombinant ComP proteins (**A**) Schematic diagram of BaComP recombinant plasmids truncated at different positions. The HisKA domain and HATPase_c domain are represented in yellow and green boxes, respectively. (**B**) SDS-PAGE of purified ComP_L_. (**C**) SDS-PAGE of purified ComP_S_. (**D**) Circular dichroism spectra of ComP_S_ in the absence and presence of ATP.

The results of structure prediction suggested that BaComP was a multispan membrane protein with a sizeable cytoplasmic region. Because of the difficulty in expressing and purifying the transmembrane proteins, we decided to study the cytoplasmic region of BaComP instead of the full-length ComP protein. Initially, we tried to express and purify the recombinant ComP_L_ (residues 382-763) protein based on the results of the prediction of secondary structure (Figure 4A). The purified protein showed a main band corresponding to the ComP_L_ protein, with an estimated molecular weight of 44 kDa, along with some contaminating bands (Figure 4B). To verify the kinase activity of ComP_L_, we measured the activity using the Kinase-Glo® Luminescent Kinase Assay [49]. The luminescent signal was directly correlated with the amount of ATP and inversely correlated with the amount of kinase activity. As expected, the reduction in luminescent signal was associated with an increasing concentration of ComP_L_ (Supplementary Figure S6). These results suggested that the purified ComP_L_ was of low purity but exhibited kinase activity.

Based on the experiments, we observed that ComP_L_ was unstable and precipitated quickly; thus, improving its purity by further purification was unfeasible. A previous study reported that proteins contain separate domains or substantially disordered regions showing dynamic variability, which affects protein stability [74]. The predicted structure of the cytoplasmic domain of ComP suggested that it consisted of several domains, which may influence its stability (Figure 2C). To improve the stability and purity of the protein, we constructed recombinant plasmids of BaComP that were truncated at different positions, including recombinant ComP_M-1_ (residues 508-763), ComP_M-2_ (residues 513-763), ComP_M-3_ (residues 518-763), ComP_M-4_ (residues 548-763), and ComP_S_ (residues 563-763) (Figure 4A). Four of the recombinant proteins mentioned above (ComP_M-1_, ComP_M-2_, ComP_M-3_, and ComP_M-4_) were not successfully expressed (data not shown). Nevertheless, the purified ComPs exhibited a single band between 25 and 30 kDa (this was consistent with the expected molecular weight of 26.3 kDa, which was calculated from the amino acid sequence), based on the 15% SDS-PAGE analysis, in which a purity level of >95% was reached (Figure 4C). These results also suggested that the histidine site of BaComP was not located in the missing region (549-562). To verify the secondary structure and folding properties of ComP_S_, we determined its structural integrity using CD spectroscopy and observed a positive band at 195 nm and two negative bands at 222 and 208 nm (Figure 4D). This result suggested that the secondary structure of ComP_S_ was properly folded. To examine whether the binding of ATP alters ComP_S_ structures, we performed the CD of ComP_S_ in the presence of ATP and calculated the percentage composition of helix, antiparallel, parallel, β-turn, and unordered structures using the software package CDPro. The results suggested that ComP_S_ was composed of 5.58% helix, 17.71% antiparallel, 15.62% parallel, 17.22% β-turn, and 43.04% unordered structures. The ComP_S_ in the presence of ATP was composed of 9.96% helix, 14.94% antiparallel, 13.75% parallel, 17.63% β-turn, and 42.13% unordered structures. We found that there was a 2.77%, 1.87%, and 0.91% decrease in antiparallel, parallel, and unordered structures, respectively. In addition, there was a 4.38% and 0.41% increase in helix and β-turn, respectively. The spectral changes indicated conformational rearrangement proceeding through biophysical interactions. ATP binding is known to enhance the thermal stability of kinases and could increase the melting temperature (*T*m) [75,76]. The Tm values of ComP_S_ in the absence and presence of ATP were detected using a Chirascan II spectropolarimeter, and the results are shown in Supplementary Figure S7 suggested that ATP induced significant changes in *T*m, and the *T*m of ComP_S_ increased 7.6°C upon ATP binding. These results indicated that ComP_S_ is thermodynamically more stable by ATP binding and that ATP stabilizes the ComP_S_ structure.

### ComP_S_ is a bifunctional enzyme with both histidine kinase and phosphotransferase activities

**Figure 5:**
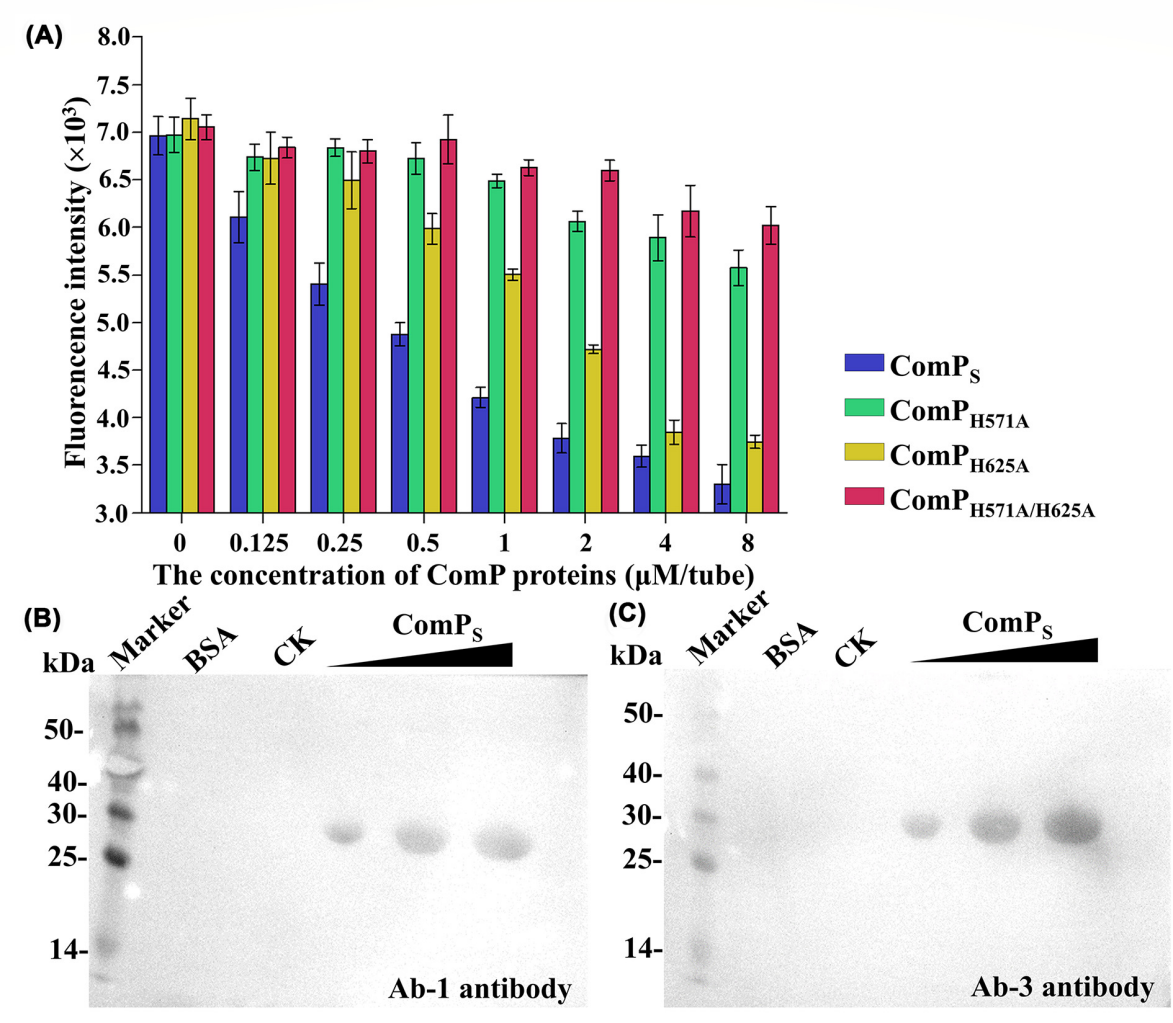
Identification of the histidine kinase activity of ComPS (**A**) Identification of the kinase activity of ComP proteins detected using the Kinase-Glo® Luminescent Kinase Assay Kit. (**B**) Western blot analysis using an Ab-1 antibody. (**C**) Western blot analysis using an Ab-3 antibody.

To verify the kinase activity of ComP_S_, a Kinase-Glo® Luminescent Kinase Assay was used [49]. It was observed that the decline in luminescent signal was associated with the increasing concentration of ComP_S_ (Figure 5A). These results indicated that ComP_S_ exhibited kinase activity (Figure 5A). In our previous study, we synthesized four kinds of pHis polyclonal antibodies (Ab-1, Ab-2, Ab-3, and Ab-4), which consisted of four haptens (A, B, C, and D) with different spacer arm lengths [48]. According to testing results, these polyclonal antibodies could specifically recognize phosphorylated histidine kinases; thus the antibodies are a powerful tool for detecting kinase activity [48]. In the present study, we further confirmed the kinase activity of ComP_S_ by Western blotting using two types of pHis antibodies (Ab-1 and Ab-3). The Western blot results revealed that both antibodies selectively recognized phosphorylated ComP_S_ but did not recognize the negative control, including BSA and unphosphorylated ComP_S_ (Figure 5B and 5C). These results demonstrated that the purified ComP_S_ exhibited kinase activity.

Previous studies have reported that typical HKs can undergo ATP-dependent autophosphorylation at a conserved His residue in the H-box, which is conserved in various species of bacteria [61,67,68]. In the present study, we conducted both sequence- and structure-based analyses of BaComP and found two crucial histidine residues (His571 and His625) in the HisKA domain, which might play an essential role in autophosphorylation. To evaluate the specific contribution of each residue, we constructed single-point and double-mutant expression vectors and then expressed and purified the mutant proteins, including ComP_H571A_, ComP_H625A_, and ComP_H571A/H625A_. The kinase activity of the mutant proteins was detected using the Kinase-Glo® Luminescent Kinase Assay [49] and was compared with the ComP_S_ wild-type. The results indicated that the decline in the luminescent signal was associated with an increasing concentration of ComP_H625A_, suggesting that ComP_H625A_ exhibited kinase activity (Figure 5A). Notably, the luminescent signal was nearly independent of the concentration of ComP_H571A_ and ComP_H571/H625A_, indicating that the mutation of His571 eliminated the kinase activity of ComP_S_ (Figure 5A). In summary, the mutagenesis study suggested that His571 played an obligatory role in autophosphorylation.

**Figure 6:**
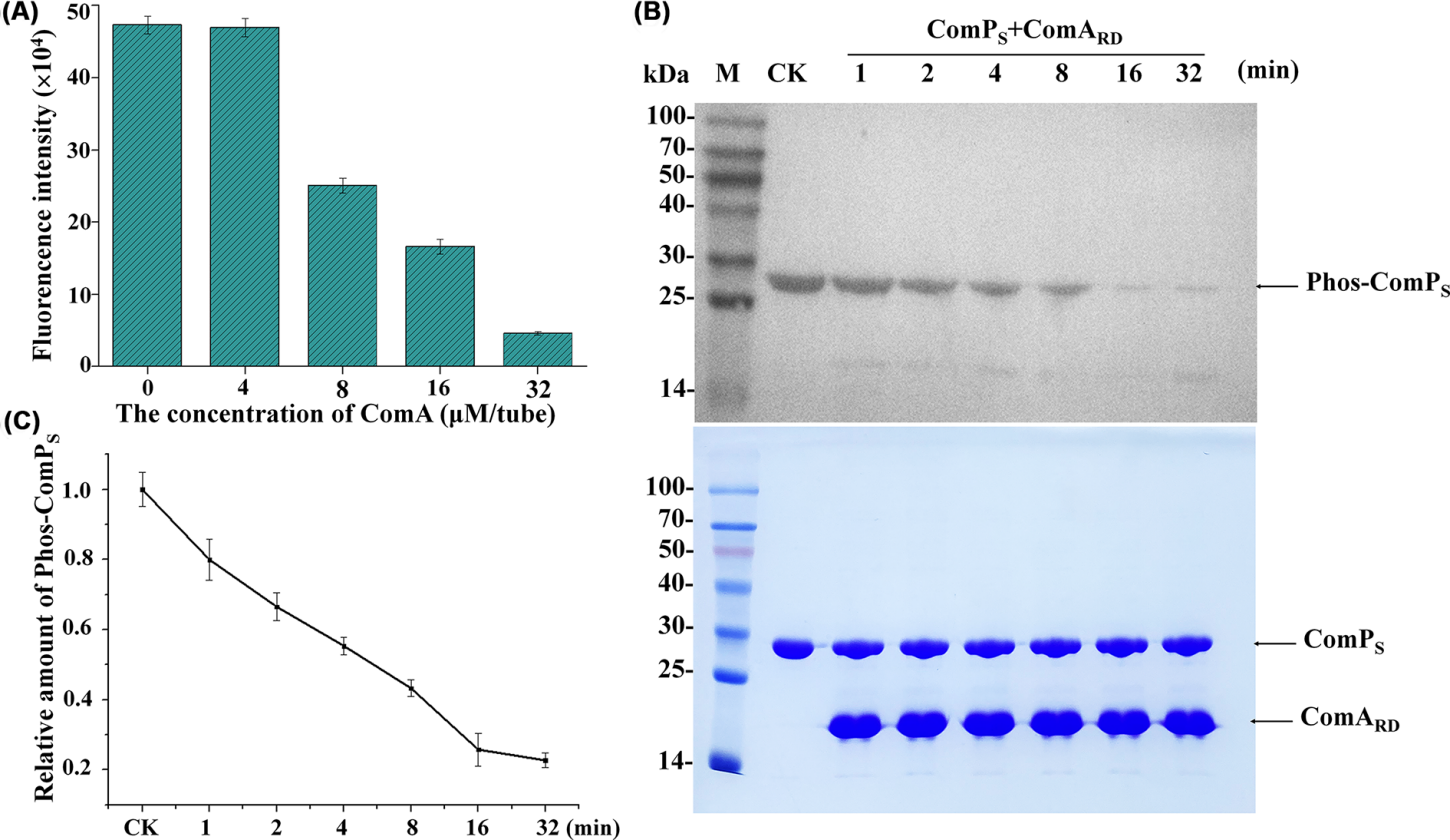
Identification of the phosphotransferase activity of ComPS (**A**) Identification of the phosphotransferase activity of ComP_S_ detected using the Kinase-Glo® Luminescent Kinase Assay Kit. (**B**) The phosphotransferase activity of ComP_S_ toward the receiver domain of ComA (ComA_RD_). CK, which was used as a negative control, indicates the initial phosphorylated ComP_S_ without the addition of ComA_RD_. Phosphorylated ComP_S_ is shown in the top panel, and the total protein controls are shown in the bottom panel. The phosphotransferase activity was quantified by the reduction in the phos-ComP_S_ relative to the amount of CK. (**C**) The relative amount of phosphorylated ComP_S_ was quantified by the reduction in the phos-ComPS relative to the amount of CK. The data are reported from *n* = 3 independent experiments.

The typical HKs autophosphorylate a histidine and then catalyze the transfer of the phosphoryl group to the response regulator [77,78]. To verify whether ComP_S_ could phosphorylate the response regulator ComA, we measured the phosphotransferase activity of ComP_S_ using the Kinase-Glo® Luminescent Kinase Assay [49]. In the reaction system, the ComP_S_ was completely autophosphorylated by adding sufficient ATP. If ComP_S_ could transfer the phosphoryl group to ComA, the histidine sites of ComP_S_ would further accept the phosphoryl group from the ATP molecule, causing a decrease in the luminescent signal. As expected, the results indicated that a decline in the luminescent signal was associated with the increasing concentration of ComA. These results suggested that the ComP_S_ protein exhibited phosphotransferase activity and could transfer the phosphoryl group to ComA (Figure 6A). To further confirm the phosphotransferase activity of ComP_S_, we performed Western blot analysis on ComA after incubation with ComP using an anti-thiophosphate ester antibody. The molecular weights of ComP_S_ and ComA were too close to separate, so we chose the receiver domain of ComA (ComA_RD_) instead of ComA. The phosphatase activity was quantified by the reduction in the phos-ComP_S_ relative to the amount of CK. Based on the results shown in Figure 6B,C, we found that the decline in the amount of phos-ComP_S_ was associated with increasing reaction time. The results suggested that ComP_S_ exhibited phosphotransferase activity and could transfer the phosphoryl group to ComA_RD_. To verify the biophysical interaction between ComP_S_ and ComA, we performed a microscale thermophoresis (MST) experiment, which is popularly used to quantify the interaction between molecules, such as proteins and small molecules. The MST results shown in Supplementary Figure S8 suggested that ComP_S_ could bind to ComA, and the binding curve yielded a *K*_d_ of 77.96 µM, with a signal-to-noise ratio of 28.35. Subsequently, we performed CD of ComA in the absence or presence of ComP_S_, and the results are shown in Supplementary Figure S9. The individual CD spectra of ComP_S_ and ComA are shown in Supplementary Figure S9A. The theoretical spectrum of ComA in the presence of ComP_S_ shown in Supplementary Figure S9B was the average of the two proteins detected in Supplementary Figure S9A. We found that there was a 1.17% and 1.62% decrease in antiparallel and parallel structures, respectively. In addition, there was a 1.73%, 0.21%, and 1.66% increase in helix, β-turn and unordered structures, respectively. The actual CD spectrum shown in Supplementary Figure S9B was not consistent with the theoretical CD spectrum, suggesting that the conformational change occurred. Collectively, the spectral changes indicated the biophysical interaction between ComP_S_ and ComA, which was consistent with the MST results.

## Discussion

The function of a protein depends on its 3D structure, but protein structures can be complex to determine experimentally. Currently, the main techniques used to determine protein structures are nuclear magnetic resonance (NMR) [79], X-ray crystallography (X-ray) [80], and cryo-electron microscopy (cryo-EM) [81]. However, the experimental approach can be long and challenging, and success is not guaranteed. In particular, the crystallization of membrane proteins such as BaComP is complicated, making it challenging to determine its 3D structure. In the present study, we predicted the tertiary structure of full-length BaComP using the program AlphaFold2. The results suggested that BaComP consisted of the N-terminal region and the C-terminal region. In the N-terminal region, BaComP contained ten TMSs, and the extracellular sensor region located at the first and second transmembrane helices was the PDZ domain, which might play a crucial role in the binding of the signal peptide ComX. In the C-terminal region, we observed two functional domains, the HisKA domain and the HATPase_c domain. The HisKA domain contained two putative histidine sites (His571 and His625), which might play an essential role in autophosphorylation. The molecular docking results suggested that the HATPase_c domain of BaComP could bind to the ATP molecule and that ATP was stabilized by eight residues from the putative N-box, G1-box, and G2-box. In the crystal structure of other HKs, the ATP or ADP molecules were located at the ATP-binding pocket and were coordinated by residues from the conserved boxes [70–72]. These results indicated that the conserved N-box, G1-box, and G2-box in BaComP played crucial roles in ATP binding.

Several assays that detect kinase activity, especially biochemical activity assays, have been developed [48,49]. Most classical kinase assays utilize radioactive reagents for detection [82]. However, radioactive isotopes damage human health and the environment, and experiments are complex and expensive. In addition, some nonradioactive and heterogeneous assays, such as enzyme-linked immunosorbent assay (ELISA) [83], reverse-phase high-performance liquid chromatography (RP-HPLC) [84,85], and nuclear magnetic resonance (NMR) have been developed [86]. Furthermore, nonradioactive homogeneous assays, such as the Kinase-Glo® Luminescent Kinase Assay [49] and the Western blot technique, have become more popular due to the low chances of contamination and convenient operational procedure. In the present study, we used the Kinase-Glo® Luminescent Kinase Assay and the Western blotting method to detect the histidine kinase activity and phosphotransferase activity of BaComP_S_. These results were similar to those of homologous proteins, and most typical HKs were bifunctional enzymes [8,87–90]. Previous studies have reported that the histidine site in the H-box is located at the first long α-helix in the HisKA domain and that HKs can autophosphorylate at the histidine site [61,67,68]. Based on the analysis of the 3D structure and sequence of BaComP, we found that two putative histidine sites (His571 and His625) were involved in two putative H-boxes, which were located at the first and second long α-helices, respectively (Supplementary Figure S4). Moreover, putative H-Box 1 was conserved with homologous proteins (Supplementary Figure S4). The mutagenesis study suggested that when the His571 residue was substituted with an alanine residue, the autophosphorylation of BaComP was nearly abolished. The results suggested that His571 played an essential role in the autophosphorylation of BaComP. However, the exact mechanism of autophosphorylation of BaComP remains unclear, and further studies are needed.

Our study provides techniques for future studies on HKs, such as BaComP and other membrane proteins from TCSs. The present study has also provided novel insight into the structural and functional relationship of BaComP and its homologous proteins.

## Supplementary Material

Supplementary Figures S1-S9

## Data Availability

All supporting data are included within the main article and supplementary materials.

## Abbreviations

AI: artificial intelligence
HK: histidine kinase
MST: microscale thermophoresis
QS: quorum sensing
RR: response regulator
TCS: transduction system
