# Supplementary material for: Insights into the structure and function of the histidine kinase ComP from Bacillus amyloliquefaciens based on molecular modeling
Source: Biosci Rep. 2022 Oct 28;42(10):BSR20220352. doi: 10.1042/BSR20220352 (PMC9620489; doi:10.1042/BSR20220352)
Supplement: Supplementary Figures S1-S9 [file BSR-2022-0352_supp.pdf]

Supplementary Materials:

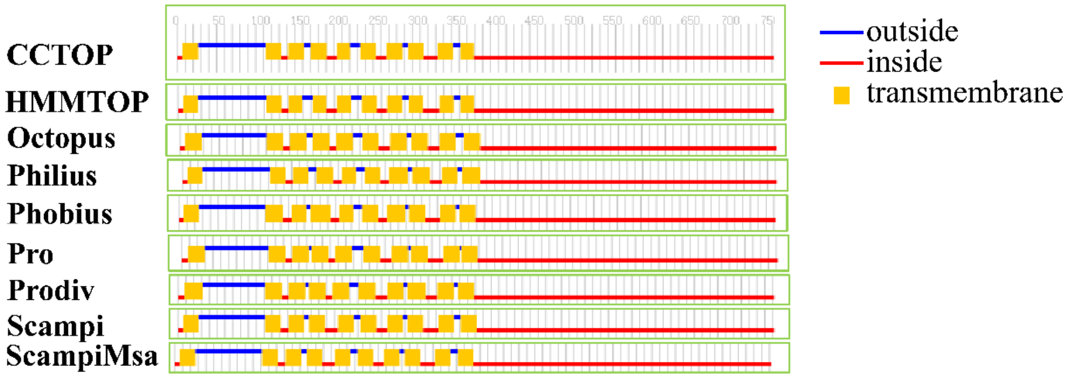

Softwares	N terminal	Numbers of TMs	PDZ domain	C terminal
CCTOP	inside	10	outside	inside
HMMTOP	inside	10	outside	inside
Octopus	inside	10	outside	inside
Philius	inside	10	outside	inside
Phobius	inside	10	outside	inside
Pro	inside	10	outside	inside
Prodiv	inside	10	outside	inside
Scampi	inside	10	outside	inside
ScampiMsa	inside	10	outside	inside

Figure S1. The secondary structure of BaComP predicted by nine different online softwares, including CCTOP, HMMTOP, Octopus, Philius, Phobius, Pro, Pro-div, Scampi and ScampiMsa.

(A)

(B)

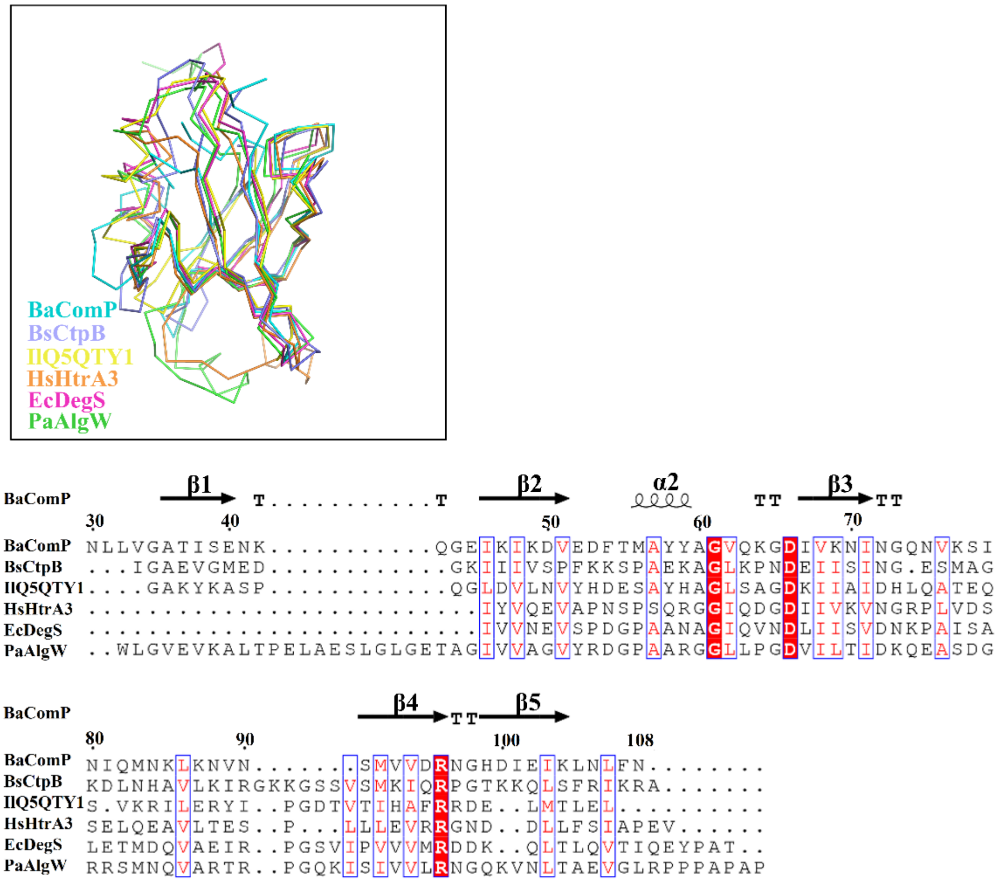

Figure S2. The overall folding and sequence of PDZ domain from various organisms. (A) The overall folding of PDZ domain from various organisms. (B) The sequence alignment of PDZ domain from various organisms. Conserved residues are shown as white letters on a red background, and similar residues are shown as red letters in blue boxes.

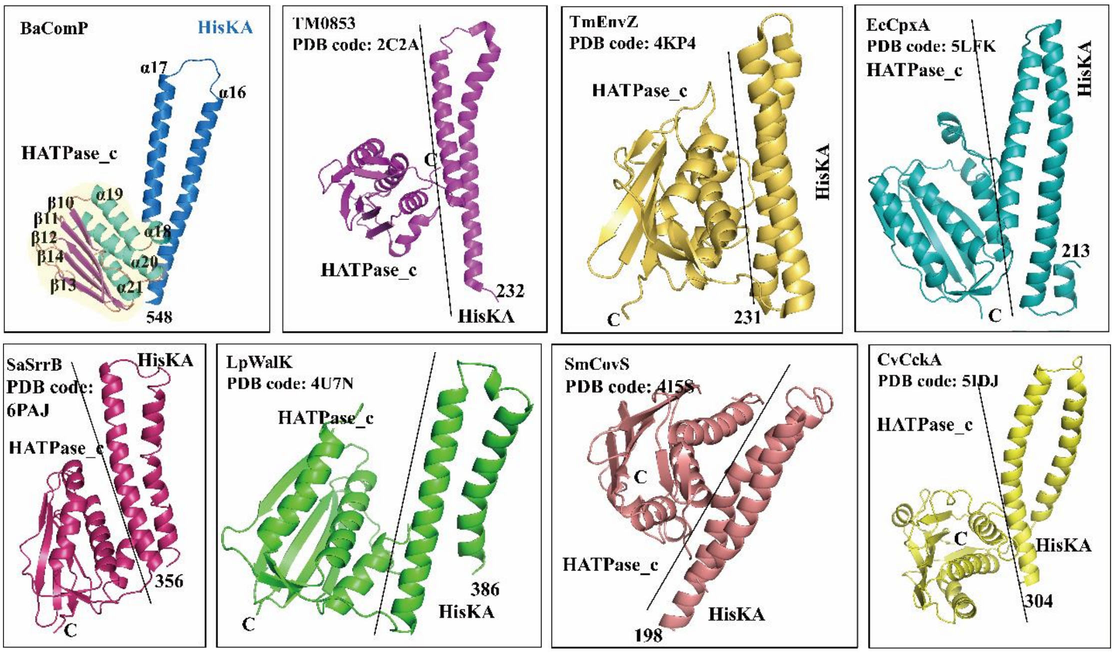

Figure S3. The overall foldings of the HisKA and HATPase_c domains from ComP and its homologous proteins. The molecules are viewed along with the cartoon.

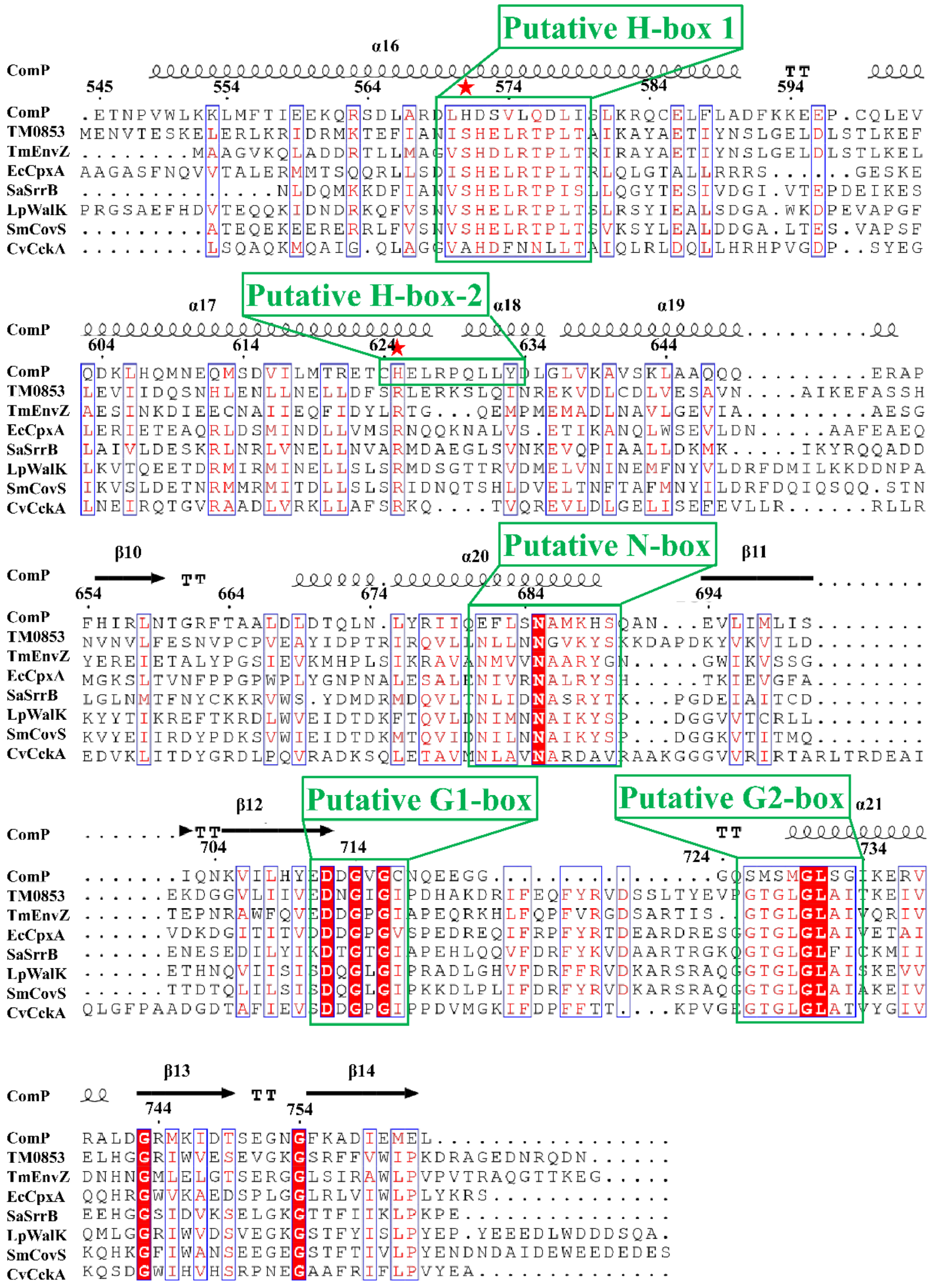

Figure S4. Sequence alignment of BaComP and its homologous proteins. Conserved residues are shown as white letters on a red background, and similar residues are shown as red letters in blue boxes.

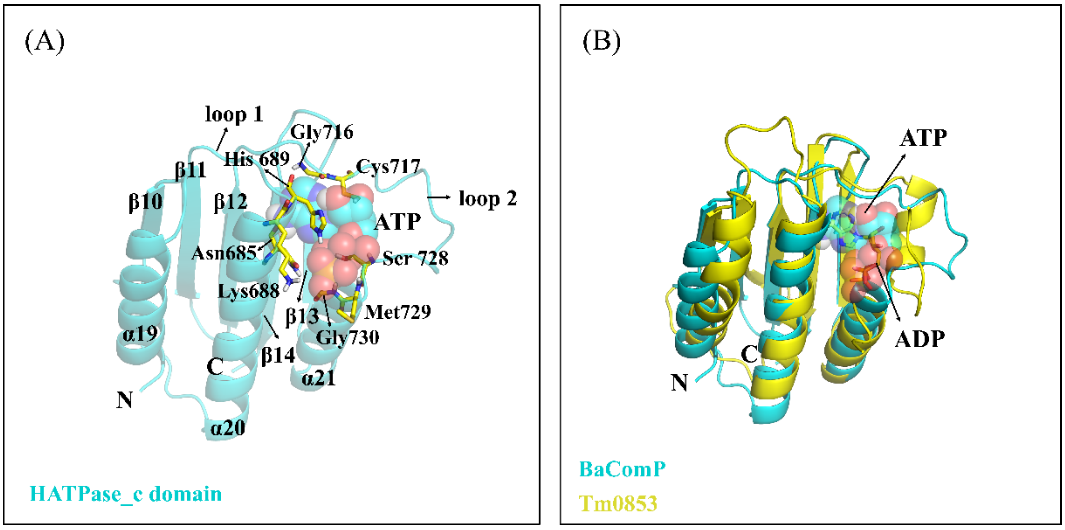

Figure S5. The ATP-binding mechanism of BaComP. (A) The ATP-binding mechanism of BaComP analyzed by HADDOCK and GROMACS. (B) The comparison of the substrate-binding pocket of BaComP and Tm0853.

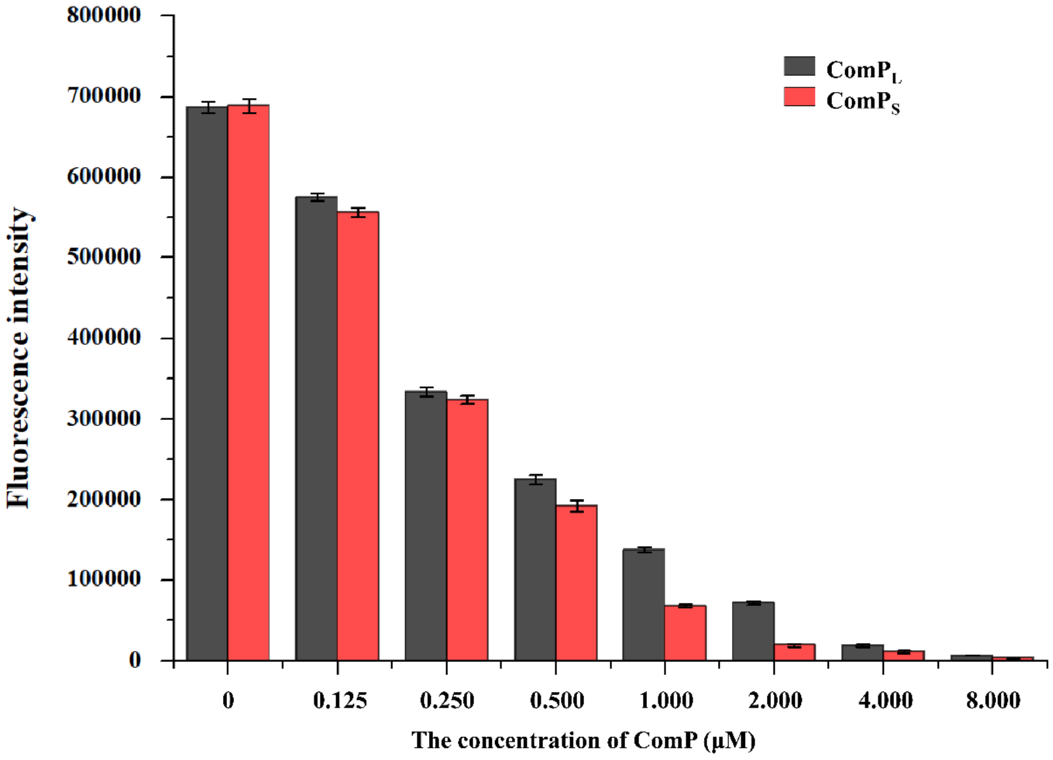

Figure S6. Identification of kinase activity of ComP$_L$ and ComP$_S$ detected by Kinase-Glo Luminescent Kinase Assay Kit.

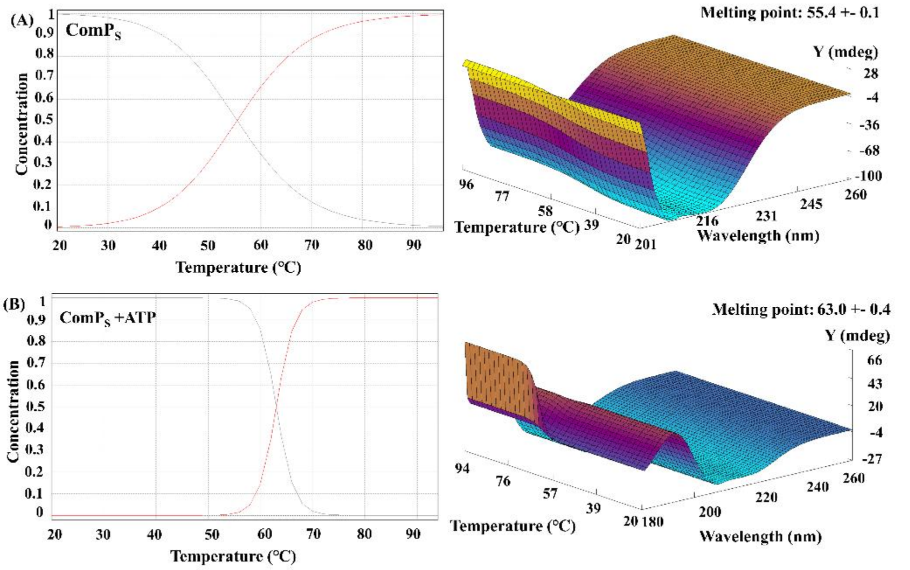

Figure S7. The Tm values of ComP$_S$. (A) The Tm value of ComP$_S$. (B) The Tm value of ComP$_S$ in the presence of ATP.

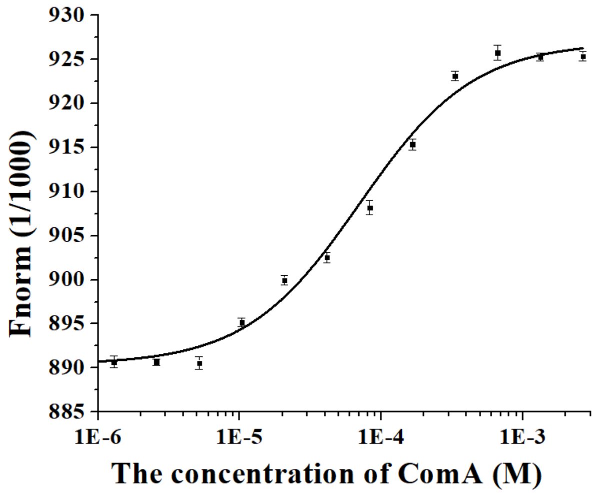

Figure S8. The binding affinity between $ComP_S$ and ComA by MST analysis.

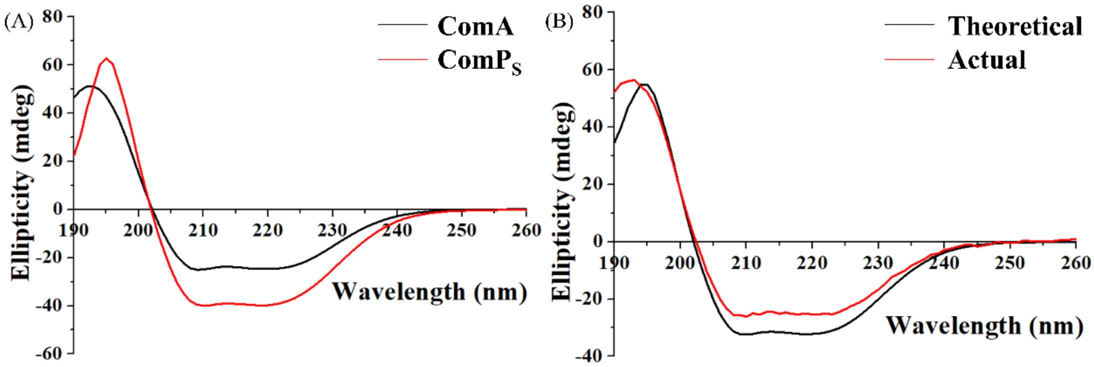

Figure S9. Interaction of ComP$_S$ with ComA by circular dichroism. (A) Individual CD spectra of ComP$_S$ and ComA. (B) Comparison of the CD spectra of ComP$_S$ incubated with ComA obtained experimentally (Actual) and the theoretically expected spectrum (Theoretical, the average of the two proteins) if no conformational change occurred.
